# Whole-genome methylation analysis reveals epigenetic variation between wild-type and nontransgenic cloned, *ASMT* transgenic cloned dairy goats generated by the somatic cell nuclear transfer

**DOI:** 10.1186/s40104-022-00764-6

**Published:** 2022-11-25

**Authors:** Hao Wu, Wendi Zhou, Haijun Liu, Xudai Cui, Wenkui Ma, Haixin Wu, Guangdong Li, Likai Wang, Jinlong Zhang, Xiaosheng Zhang, Pengyun Ji, Zhengxing Lian, Guoshi Liu

**Affiliations:** 1 National Engineering Laboratory for Animal Breeding, Key Laboratory of Animal Genetics and Breeding of the Ministry of Agricultural, Beijing Key Laboratory for Animal Genetic Improvement, College of Animal Science and Technology, China Agricultural University, Beijing, 100193 China; 2 Sany Institute of China Agricultural University, Sanya, 572025 China; 3 Institute of Animal Husbandry and Veterinary, Academy of Agricultural Sciences of Tianjin, Tianjin, 300192 China; 4 Qingdao Senmiao Industrial Co., Ltd., Qingdao, 266101 China

**Keywords:** Acetylserotonin-O-methyltransferase, Dairy goat, DNA methylation, Gene editing, Somatic cell nuclear transfer

## Abstract

**Background:**

SCNT (somatic cell nuclear transfer) is of great significance to biological research and also to the livestock breeding. However, the survival rate of the SCNT cloned animals is relatively low compared to other transgenic methods. This indicates the potential epigenetic variations between them. DNA methylation is a key marker of mammalian epigenetics and its alterations will lead to phenotypic differences. In this study, *ASMT *(acetylserotonin-O-methyltransferase) ovarian overexpression transgenic goat was produced by using SCNT. To investigate whether there are epigenetic differences between cloned and WT (wild type) goats, WGBS (whole-genome bisulfite sequencing) was used to measure the whole-genome methylation of these animals.

**Results:**

It is observed that the different mCpG sites are mainly present in the intergenic and intronic regions between cloned and WT animals, and their CG-type methylation sites are strongly correlated. DMR (differentially methylated region) lengths are located around 1000 bp, mainly distributed in the exonic, intergenic and intronic functional domains. A total of 56 and 36 DMGs (differentially methylated genes) were identified by GO and KEGG databases, respectively. Functional annotation showed that DMGs were enriched in biological-process, cellular-component, molecular-function and other signaling pathways. A total of 10 identical genes related to growth and development were identified in GO and KEGG databases.

**Conclusion:**

The differences in methylation genes among the tested animals have been identified. A total of 10 DMGs associated with growth and development were identified between cloned and WT animals. The results indicate that the differential patterns of DNA methylation between the cloned and WT goats are probably caused by the SCNT. These novel observations will help us to further identify the unveiled mechanisms of somatic cell cloning technology, particularly in goats.

**Supplementary Information:**

The online version contains supplementary material available at 10.1186/s40104-022-00764-6.

## Introduction

Sheep is one of the most important commercial livestock among others. Currently, it also serves as a biomedical animal model to produce antibodies and other bio-substances which are beneficial for human health [1]. In addition, sheep is also used as the pathological models to mimic the rare human diseases. For example, the *Cln6* sheep model is used to simulate the human Batten disease [2]. Currently, much attention has been given to the genomics of this species with the advance of the methodologies [3]. Gene editing can be used to edit sheep genome to improve their traits and production performance [4]. Since the birth of Dolly, the SCNT technology has been continuously optimized and has been applied to different fields including the medicine and agriculture [5]. For example, Deng et al. have successfully constructed a cloned sheep of *TLR4* overexpression by using SCNT to create a novel disease-resistant animal model [6]. Tao et al. have developed *AANAT* (aralkylamine N-acetyltransferase) cloned goats with elevated endogenous melatonin level and anti-inflammatory capacity [7]. In 2018, the successful cloning of *Macaca fascicularis* was a landmark achievement of SNCT technology in the clone of high rank of species including primates [8]. Although the application of SCNT technology has made great achievements as mentioned above, several shortcomings still need to be addressed. These include its relatively high cost and low success rate. Trauma during micromanipulation, inadequate reprogramming ability of oocytes, resistance to reprogramming of the used donor nuclei, and abnormalities caused by in vitro culture of reconstructed SCNT embryos can all lead to cloning failure [9]. However, the main cause of low SCNT efficiency in mammals is an incomplete reprogramming of transcriptional activity for donor cell-descended genes [10]. Incomplete or incorrect epigenetic reprogramming of epigenetic memory was found to be one of the main factors which decrease the efficiency of somatic cell cloning in goat [11]. The relatively low reprogramming efficiency of SCNT embryo often leads to the jeopardized fetal development and growth defects of the embryos [12]. Methods to improving the reprogramming efficiency of cloned embryos associated with SCNT have mainly focused on the drug intervention and genetic modification, however, few breakthroughs have been achieved [13]. As the best knowledge of ours, the reprogramming efficiency of cloned embryos is mainly regulated by the epigenetics. DNA methylation is the most important epigenetic modification. Also, DNA methylation can alter the mammalian phenotype by targeting non-coding elements of introns and action of DNA proteins [14]. For example, DNA methylation regulates gene expression by recruiting proteins involved in gene suppression or by inhibiting the binding of transcription factors to DNA [15]. Thus, investigation of the alterations of DNA methylation is a key step to understand the epigenetics of embryo development [16]. SCNT embryos need to be reprogrammed to restore totipotency. Abnormal DNA methylation reduces reprogramming efficiency and leads to gene expression errors [17]. Eliminating abnormal DNA methylation can improve the development of embryos for somatic cell nuclear transfer and improve the cloning efficiency [18]. By use of the genome-wide methylation analysis, it is found that in the cloned or in vitro fertilization pig embryos, the abnormal reprogramming events are mainly due to insufficient DNA demethylation [19]. The methylation level and the expression of imprinted genes (*IGF2R*, *PEG3*, and *ZFP64*), and zygotic genes (*DUXA*, *IGF2BP1*, *WT1*, and *ZIM3*) are associated which suggests that DNA methylation is in the tight control of ZGA (zygotic genome activation) by regulating the expression of the critical genes [20]. In this study, we will generate the *ASMT* overexpressed dairy goats by using CRISPR/Cas9 at the FGF5 site. ASMT is the rate-limiting enzyme of melatonin synthesis and its overexpression increases the endogenous melatonin production in goats [21]. The genome-wide methylation analysis will be performed to both the *ASMT* overexpressed and WT dairy goats. The purpose is to uncover the characteristics of epigenetic differences caused by cloning technology. The results will provide some insights for the cloning mechanisms.

## Materials and methods

### Animals

A total of 158 dairy goats were used in this study, including 107 donors and 51 recipients. Donor and recipient dairy goats weighed 55–60 kg and were healthy without the reproductive disorders.

### Expression vector construction of the *ASMT* gene

The eukaryotic expression vector of GDF9-ASMT was obtained from Dr. He Changjiu (Laboratory of Huazhong Agricultural University, Wuhan, China) (Fig. S1). The vector sequence 5-HA was synthesized and 5′ (AatII) and 5'UTR (KpnI) and 3'UTR ([]) and 3′ (MluI) loci were added to both ends of the 5-HA sequence. 5-HA was cloned into vector GDF9-ASMT by 5′ AatII and 3′ MluI. The vector sequence 3-HA was synthesized and 5′ (PciI) and 5'UTR ([]) and 3'UTR (KpnI) and 3′ (PciI) loci were added to both ends of the 5-HA sequence. 3-HA was cloned into vector 5-HA in GDF9-ASMT by 5′ PciI and 3′ PciI to construct vector 3-HA-GDF9-ASMT-5-HA.

### Donor cell preparation

Male primary fetal fibroblast cell lines of dairy goats (*Capra aegagrus hircus*) were selected for the studies. The cells with normal growth status were collected for electrotransformation. Cas9-sgRNA and 3-HA-GDF9-ASMT-5-HA vectors were electrically transfected into cells using an electrotransferring apparatus (Nucleofector II, Lonza, Koln, Germany). Single cell sorting was performed on a BD ARIA3 cell sorter (Becton Dickinson, San Jose, CA, USA) 48 h after electrotransferring. Single cell clones with normal growth status were selected after about 2–3 weeks. One-third of the single-cell clones were left in the original pore and two-thirds were digested for extracting genomic DNA. High-fidelity enzyme (GXL-R051A, TAKARA, Beijing, China) was used for identification of the genotypes. The primers were shown in Table 1.

**Table 1:** GDF9-ASMT primers

Primer	Sequence (5′ to 3′)	Length, bp	TM, °C
ASMT-3HA-F	TTCCTTGACCCTGGAAGGTG	1487	58
ASMT-5HA-R	AGTTTCCATGTCACATAGCCA	1487	58
ASMT-5HA-F	AACCTCTCATCACCTGATCAC	1500	59
ASMT-5HA-R	AAGGAGGTACAGCTGAGACTAA	1500	57

**Table 2:** Analyze software models

Analysis	Software or database	Version
Data QC	Cutadapt [22]	Version 1.9.1
Mapping	Bismark [23]	Version 0.7.12
Differeantial mCpG sites	MethylKit [24]	Version 3 0.7.12
Detection and annotaion	Samtools [25]	Version1.6
	Annovar [26]	Version 21 Feb 2013
DMR	swDMR [27]	Version 1.0.7
	Annovar [26]	Version 21 Feb 2013
Enrichment analysis	GO Database [28, 29]	
	KEGG Database [30, 31]	Version 03/21/2011

addresses

• GO DataBase: http://geneontology.org/

• KEGG DataBase: http://www.genome.jp/kegg/kegg1.html

• Bismark: http://www.bioinformatics.babraham.ac.uk/projects/bismark/

• MethylKit: https://github.com/al2na/methylKit

• swDMR: https://sourceforge.net/projects/swdmr/

### Concurrent estrus and superovulation

The healthy donors and recipients of the ewes were treated with simultaneous ovulation and timing insemination. The donors and recipients were implanted with CIDR (Eazi-Breed® CIDR® Sheep and Goat Device Pfizer Animal Health, New Zealand) for 16 d in advance. From 84 h before the withdrawal of the CIDR, 240 IU FSH (Ningbo Hormone Products Co., Ltd., Ningbo, China, 110044648) was injected every 12 h according to body weight. 38 h after withdrawal of CIDR, 100 IU LH was injected (Ningbo Hormone Products Co., Ltd., 110044634). The CIDR was removed from donors and recipients at the same time. The 250 IU PMSG (Ningbo Hormone Products Co., Ltd.,110044564) was injected into the recipients. 12 h after the withdrawal of CIDR, donor and recipient ewes were subjected to a test with a test ram. Feeding was stopped 12 h before surgery and ovulation and luteal conditions were checked about 60 h after CIDR withdrawal. The oocytes were rushed out from the fallopian tubes and the high-quality oocytes were selected and recorded under stereoscope.

### SCNT and pregnancy diagnosis

Mature oocytes were incubated in TCM199 (Earle salts; Gibco/Life Technologies Inc., New York, USA, 12340) with 5 μg/mL CB (Sigma-Aldrich, St. Louis, MO, USA, C6762) and 5 μg/mL Hochest 33,342 (Sigma-Aldrich, 14533) for 5–10 min. The oocytes (OLYMPUS, ON3, Tokyo, Japan) were denucleated with a micromanipulator and the donor cells were injected into the denucleated oocytes. After half an hour, electric fusion was performed. The fusion was first balanced in the fusion solution for 3–5 min and then placed into a fusion tank covered with the fusion solution (BTX Inc., San Diego, CA, USA). The oocyte pole body was adjusted parallel to the electrode and a DC pulse was applied 2 direct current pulses of 2.0 kV/cm for 25 μs using an ECM2001 Electrocell Manipulator (BTX Inc., SanDiego, CA, USA). 30 min later, the unfused eggs were undergoing a second fusion. The reconstructed embryos were incubated in TCM199 containing 10 μg/mL of actinomycetes CHX (Sigma-Aldrich, C7698) and 5 μg/mL of CB for 4–5 h. Wash with developmental solution for 3–5 times, the embryos were transferred to the developmental solution for overnight culture and transplanted at the next day. The pregnancy of the recipient ewes was examined about 60 d after embryo transplanted.

### Genome-wide methylation analysis

#### DNA methylation sequencing by Illumina

DNA methylation sequencing by Illumina HiSeq Next generation sequencing library preparations was constructed following the manufacturer’s protocol. For each sample, 1 μg genomic DNA was randomly fragmented to < 500 bp by sonication (Covaris S220). The fragments were treated with End Prep enzyme mix for end repairing, 5'-Phosphorylation and dA-tailing in one reaction, followed by a T-A ligation to add methylated adaptors to both ends. Size selection of adaptor­ligated DNA was then performed using beads, and fragments of ~ 410 bp (with the approximate insert size of 350 bp) were recovered. Then bisulfite conversion was performed using the related kit. Each sample was then amplified by PCR using P5 and P7 primers, with both primers carrying sequences which can anneal with flow cell to perform bridge PCR and P7 primer carrying index allowing for multiplexing. The PCR products were cleaned up using beads, validated using an Qsep100 (Bioptic, Taiwan, China), and quantified by Qubit 3.0 Fluorometer (lnvitrogen, Carlsbad, CA, USA).

Then libraries with different indices were multiplexed and loaded on an Illumina HiSeq/Novaseq instrument according to manufacturer’s instructions (Illumina, San Diego, CA, USA) or a MGl2000 instrument according to manufacturer’s instructions (MGI, Shenzhen, China). Sequencing was carried out using a 2 × 150 paired-end (PE) configuration; image analysis and base calling were conducted by the HiSeq Control Software (HCS) + OLB + GAPipeline-1.6 (Illumina) on the HiSeq instrument, image analysis and base calling were conducted by the NovaSeq Control Software (NCS) + OLB+ GAPipeline-1.6 (Illumina) on the NovaSeq instrument, image analysis and base calling were conducted by the Zebeacall on the MGl2000 instrument.

#### Biological information analysis

- ① The bcf2fastq (Version 2.17.1.14) was used for image Base calling to obtain the original Data (Pass Filter Data, PF) of the sequencing samples. Sequencing data was stored in FASTQ format.
- ② Cutadapt (Version 1.9.1) was used to remove connectors and low-quality sequences from the raw Pass Filter Data to obtain Clean Data for subsequent information analysis.
- ③ Bismark (Version 0.7.12) was used to alignment clean data to reference genome sequence [*Capra hircus ARS1*] (https://www.ncbi.nlm.nih.gov/genome/?term=goat). The reference sequence reads, genome coverage and average depth were statistically compared.
- ④ Bismark (Version 0.7.12) was used to compare the methylation sites. The average methylation rate of methylation sites within a specific window length was calculated by windowing and the genome-wide distribution map was drawn.
- ⑤ According to the results of genome-wide methylation sites, all mCpG type sites were extracted. The coverage of each locus and the methylation rate of different genes were calculated and the depth distribution map was drawn.
- ⑥ Based on the methylation rate information of mCpG sites, Pearson correlation test was conducted among all samples to evaluate the correlation of each sample.
- ⑦ Functional annotations were performed for the differential mCpG sites, and the number of differential methylation sites in each functional region was counted and the distribution map was drawn.
- ⑧ SwDMR software was used for regional identification of DMR between samples. The software uses a window method with size of 1000 bp to make statistics of methylation regions between samples or between groups and to identify methylation regions with significant differences. The length distribution of DMR region was calculated and the density distribution map was drawn.
- ⑨ ClusterProfiler (R Package, Version:3.8.1) was used for GO term/KEGG pathway enrichment analysis of differential DMR genes.

### Database and software

The database and software used in this study are shown in Table 2.

### Statistical analysis

Data were expressed as mean ± standard error. One-way analysis of variance was performed followed by Duncan's test with SPSS software, Version 25.0 (IBM SPSS Statistics, Armonk, NY, USA). *P* < 0.05 was considered statistically significant.

## Result

### GDF9-ASMT vector construction and target strategy

**Fig. 1:**
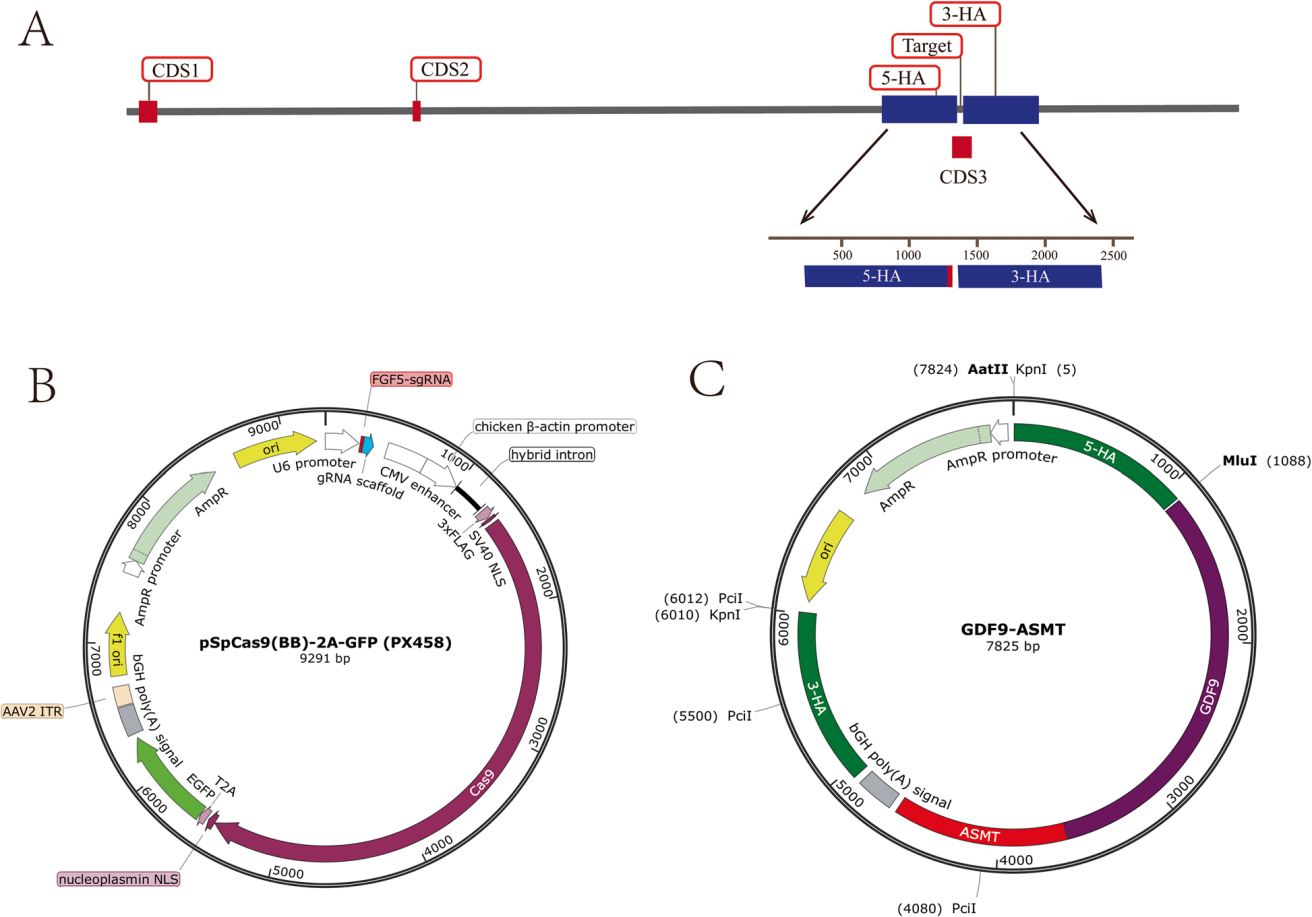
Construction of *FGF5* target system. **A** Goat *FGF5* site targeting strategy; **B** FGF5-sgRNA-Cas9 vector; **C** GDF9-ASMT vector

The third exon of goat *FGF5* was used as the target site (Fig. 1A). FGF5-sgRNA sequence were donated from the laboratory of Professor Lian (Laboratory of China Agricultural University, Beijing, China). FGF5-sgRNA was connected with the unintegrated vector PX458 to form the target vector of Cas9-sgRNA (Fig. 1B). The sequence of goat 5’ homologous arm is located between the 20,006 and 21,065 bases of *FGF5* gene on chromosome 6, with a total length of 1055 bp. The sequence of the 3’ homologous arm was located between the bases 21,109 and 22,166, with a full length of 1058 bp (Fig. 1C). Cas9 vector is targeted to cut under the guidance of sgRNA and the homologous recombination occurs in the presence of homologous repair template to realize the exogenous gene knock in.

### Generation of the *ASMT* overexpressed goats

**Fig. 2:**
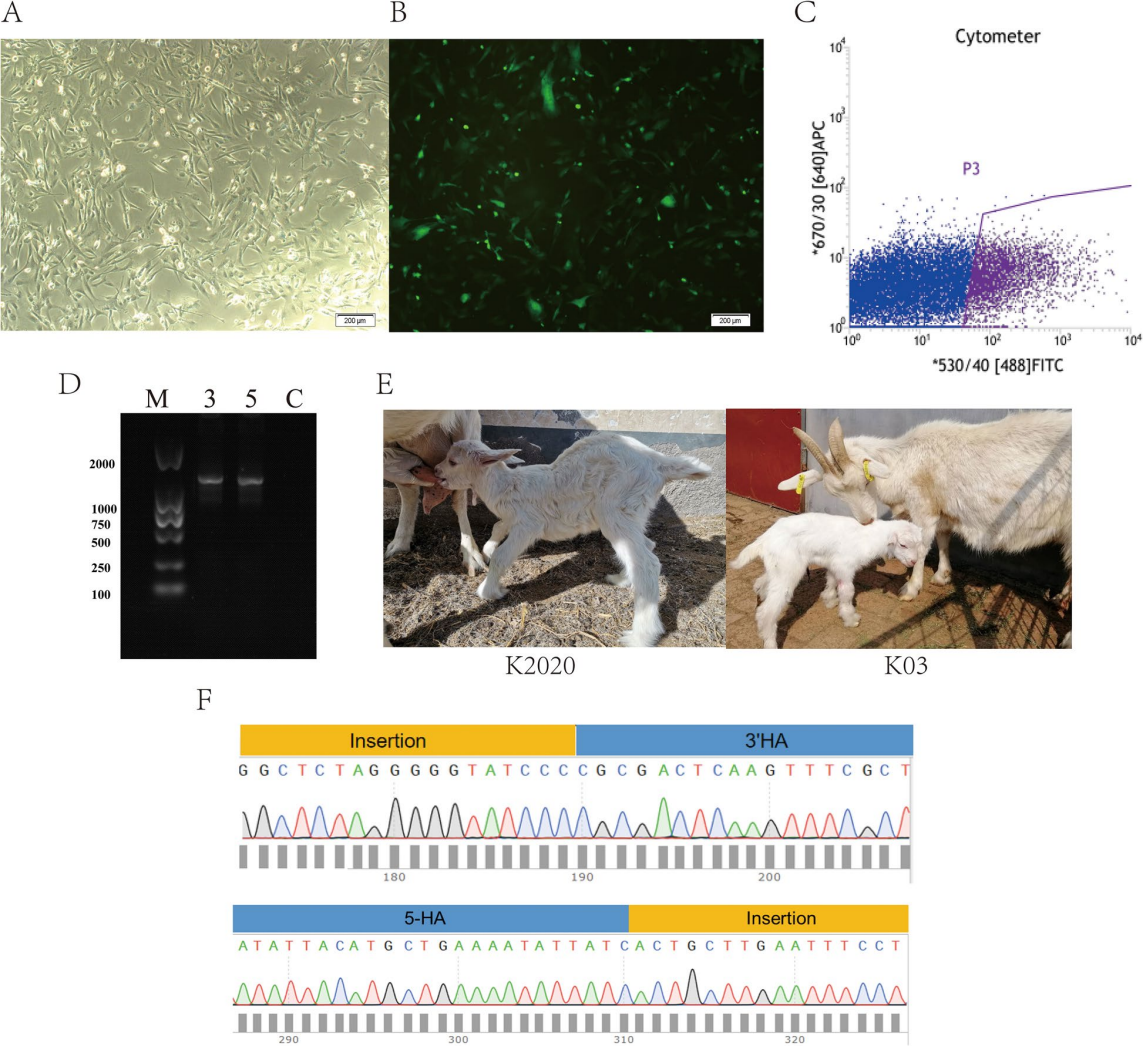
*ASMT* overexpressed goat production. **A** Cell transfection (bright field); **B** Cell transfection (fluorescence); **C** Flow cytometry sorting; **D** PCR identification; **E** Cloned goats; **F** Sequencing comparison

Goat fetal fibroblasts were transfected with Cas9 vector and GDF9-*ASMT* donor vector using A033 transfection procedure and the cell growth status was observed 48 h after transfection (Fig. 2A-B). The cells after transfection were sorted by flow cytometry analyzer and positive cells were screened for somatic cell nuclear transfer. The positive cells were identified by sequencing comparison (Fig. 2F). A total of 1008 eggs were obtained after superovulation of 107 donor ewes and 522 embryos were successfully reconstructed. 5 of the 51 transplant recipients were pregnant, and 1 of the 2 lambs was positive. The information is shown in Table 3.

**Table 3:** General table of somatic cell nuclear transfer in dairy goats

Donor goats	Oocytes	Usable oocytes	Oocytes enucleated, %	Donor cells	Reconstructed embryos	Recipients	Pregnancy	Lambing number
30	199	181	94.46% (171/181)	GDF9-ASMT/control	131	13	2	1
77	809	709	92.95% (659/709)	GDF9-ASMT	391	38	3	1

### Raw data analysis of genome-wide methylation sequencing

The cloned goats K2020, K03 and WT goat S2 were selected for whole-genome methylation sequencing. Clean data about 320 GB were obtained by bisulfite genome sequencing of the three samples. Q30 values were all greater than 87.82% (Table 4). The percentage of Clean Bases in PF Bases reached 98.82%. The quality control data were compared with the reference genome. The average comparison rate was 86.34%, the average coverage rate was greater than 107.14%, and the average coverage depth was greater than 31.98× (Table 5). These results indicate that the sequencing data is of high quality and conducive to bioinformatics analysis.

**Table 4:** Genome-wide DNA methylation quality control data

Sample	PF Reads	Clean Reads	Ratio of Reads, %	PF Bases	Clean Bases	Ratio of Bases, %	Q30, %
K2020	920,251,338	917,384,276	99.69	138,037,700,700	136,576,198,940	98.94	89.63
S2	602,531,784	6,002,777,190	99.63	90,379,767,600	89,270,005,346	98.77	89.81
K03	643,364,184	640,837,624	99.61	96,504,627,600	95,292,503,626	98.74	87.82

(1) Sample: Name of sequencing sample

(2) PF Reads: The number of original Reads

(3) Clean Reads: The number of Reads after QC

(4) Ratio of Reads: The percentage of Clean Reads in PF Reads

(5) PF Bases: The number of Bases of the original data

(6) Clean Bases: The number of Bases after QC

(7) Ratio of Bases: The percentage of Clean Bases in PF Bases

(8) Q30: Calculate the percentage of bases with Phred value greater than 30 in the total base

**Table 5:** Comparison results of reference genomic data

Sample	Total Reads	Reads mapped to genome	Mapped Reads ratio, %	Coverage, %	Mean depth	Bisulfite conversion rate, %
K2020	917,384,276	840,319,806	91.60	107.38	43.59	99.41
S2	566,928,456	434,321,980	76.61	107.26	22.23	99.42
K03	643,364,184	581,957,164	90.81	107.14	30.11	99.41

(1) Sample: Name of sequencing sample

(2) Total Reads: The number of all sequenced Reads

(3) Reads mapped to genome: Number of Reads mapped to the reference genome

(4) Mapped Reads ratio: The proportion of Reads Mapped against the reference genome

(5) Coverage: Covered Bases/Genome Bases × 100%

(6) Mean depth: The average depth of covering bases

(7) Bisulfite conversion rate: Cytosine conversion rate of Bisulfite treatment (Unmethylated/Total Cytosine*100%)

### Detection of different types of methylation in the whole-genome

**Fig. 3:**
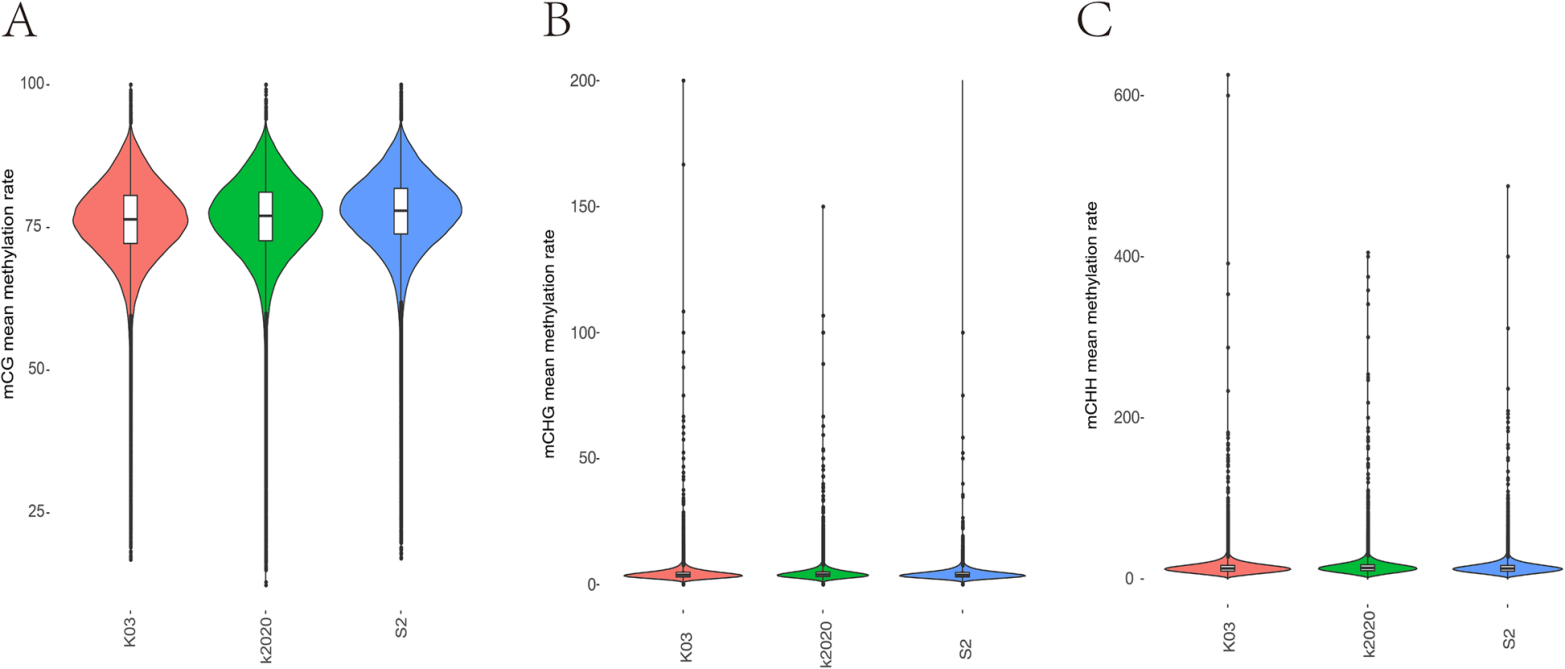
Distribution of methylation rate of the whole genome.** A** CG type; **B** the CHG type; **C** CHH type

**Fig. 4:**
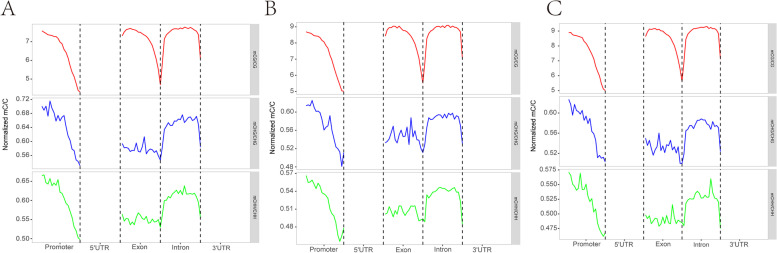
Average methylation rate of gene functional region. **A** *ASMT* transgenic cloned goat (K2020); **B** nontransgenic cloned goat (K03); **C** control goat (S2). Note: The abscissa is the classification of functional areas, and the ordinate is the mean methylation rate

**Fig. 5:**
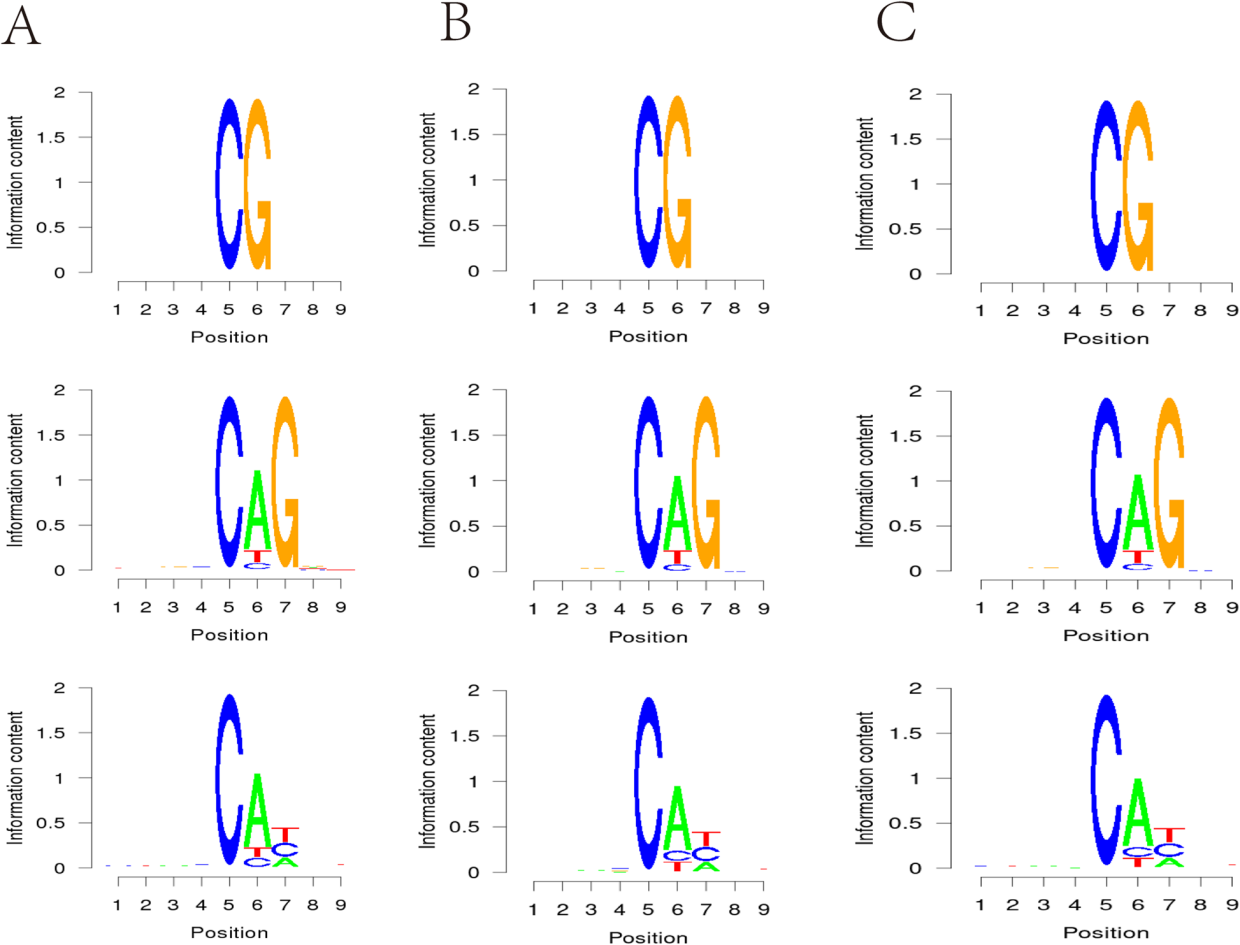
Sequence characterization of methylcytosine. **A** *ASMT* transgenic cloned goat (K2020); **B** nontransgenic cloned goat (K03); **C** control goat (S2). Note: The *X* axis represents the location of the functional areas, the coordinate position of site C is 5, and the *Y* axis represents the degree of base enrichment. Different colors represent different base types. In the order of top to bottom, the Weblogo diagram of CG methylation site, CHG methylation site and CHH methylation site is shown

The C-base sites of the whole-genome were detected and the methylation patterns of CG, CHG and CHH (H = A, T, or C) were systemically analyzed. The methylation ratios of the three samples were similar. Most of the methylations were CG types indicating that CG methylation is the dominant one among others (Fig. 3). When genes or transcripts was classified as functional regions, the methylation sites were mainly distributed in the promoter, exon and intron. The mean methylation rate of gene functional region was no significant difference (Fig. 4), nor did the methylation density (Fig. S3) among the three samples. The sequence characteristics of the base in the range of 9 bp upstream and downstream of the methylation site were counted, and the sequence characteristics of CG, CHG and CHH were significantly different between *ASMT* cloned goat and others. And the sequence characteristics of CHG and CHH among different samples were significantly different. In K2020, two side sequence of the CHH extend position site 1 and 9, respectively. The base at sites 1 and 9 is T. Also, in K2020, the middle site of CHG was A, T, C in sequence. But in K03 and S2, the middle site of CHG was A, C, T in sequence. Two side sequence of the CHG were also different (Fig. 5).

### Genome-wide analysis of mCpG sites

**Fig. 6:**
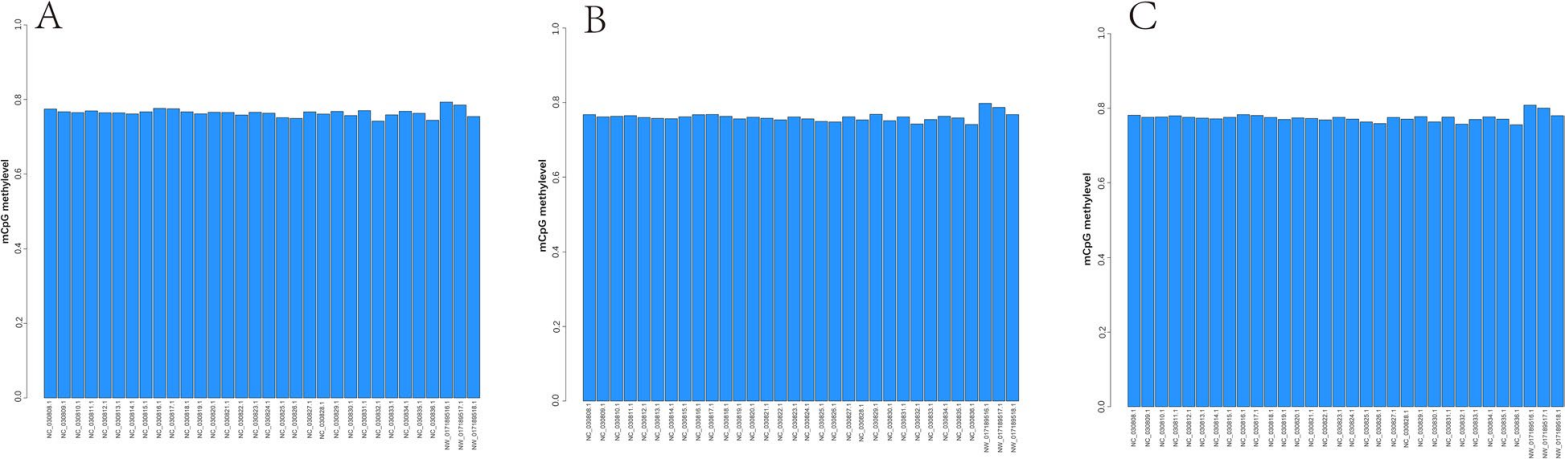
Proportional distribution of mCpG on chromosome. **A** *ASMT* transgenic cloned goat (K2020); **B** nontransgenic cloned goat (K03); **C** control goat (S2). Note: The abscissa represents each chromosome; The vertical axis represents the proportion of mCpG to total CpG loci in the chromosome

**Fig. 7:**
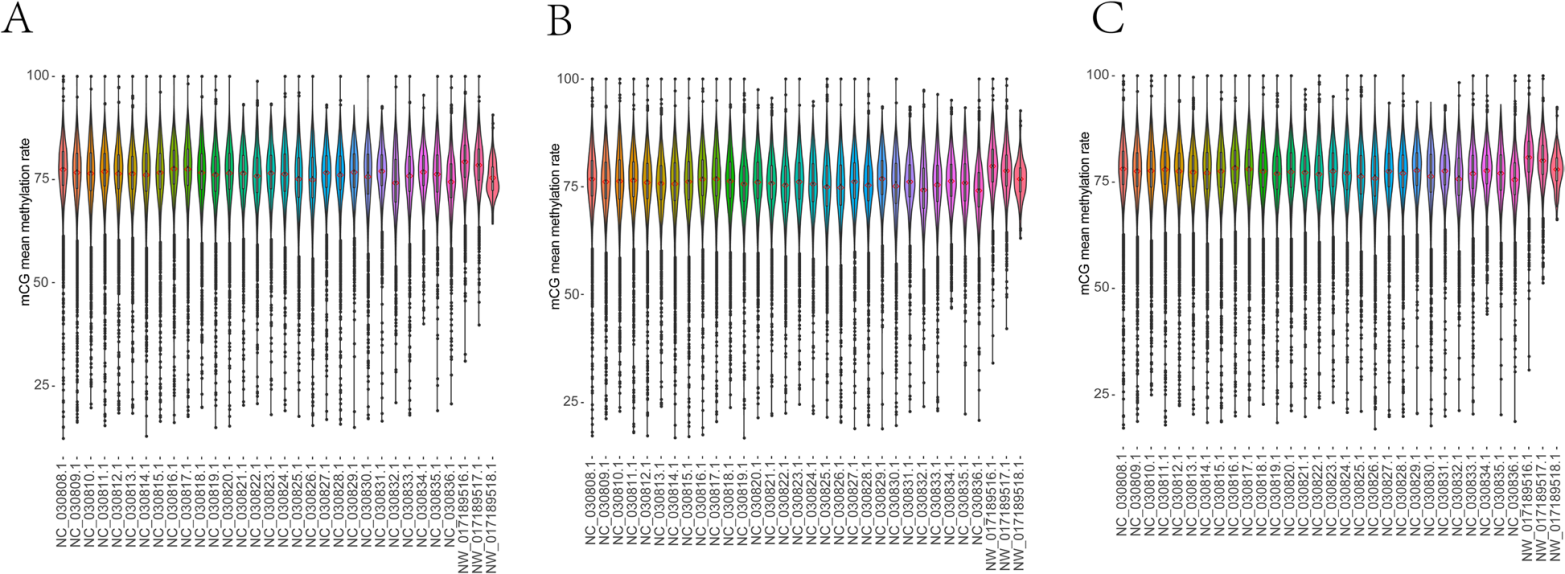
Proportional distribution of mCpG on chromosome. **A** *ASMT* transgenic cloned goat (K2020); **B** nontransgenic cloned goat (K03); **C** control goat (S2). Note: The abscissa represents each chromosome; The vertical axis represents the proportion of mCpG to total CpG loci in the chromosome

CpG methylation site is the main type of methylation in the genome, and it can reflect the status of the genome. All mCpG sites were extracted for further analysis based on the results of genome-wide methylation sites. The genome-wide depth of mCpG loci was mainly around 20× (Fig. S4). The methylation rate of mCpG sites in the three samples was more than 90% in the whole-genome (Fig. S5). There was no significant difference in the proportion of mCpG to all CpG sites on each chromosome (Fig. 6). The methylation rate distribution of mCpG on each chromosome was analyzed and the methylation rates of NC-030834.1 and NW-017189518.1 were significantly different from other chromosomes (Fig. 7).

### Differential mCpG loci detection and annotation

**Fig. 8:**
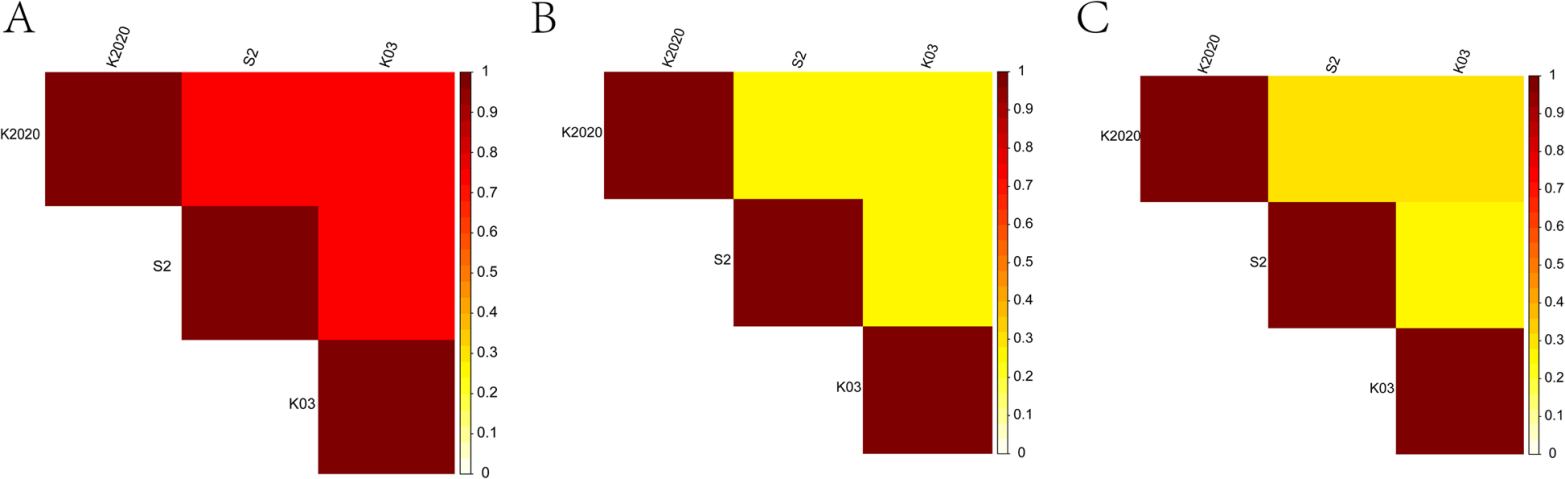
Sample correlation heat map analysis. **A** CG type; **B** CHG type; **C** CHH type. Note: the heat map between the horizontal and vertical coordinates of each two points shows the correlation between two samples

**Fig. 9:**
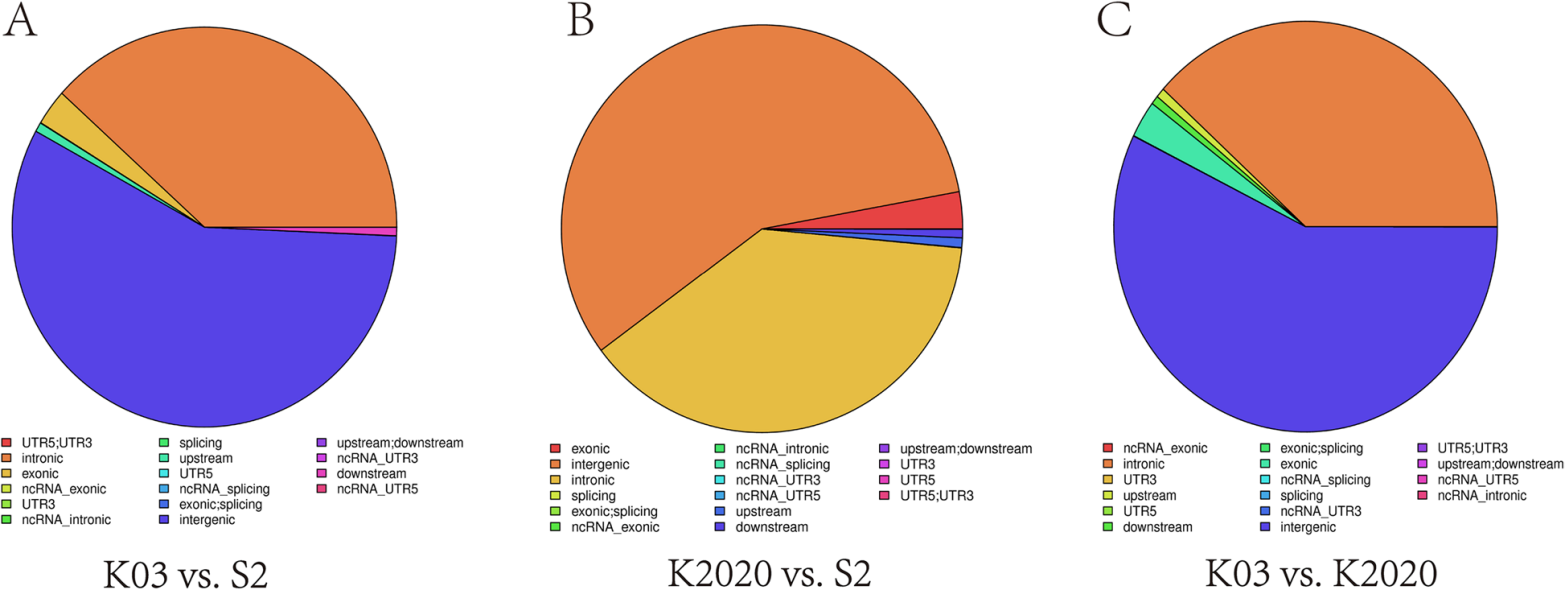
Distribution of functional regions of different methylation sites. **A** nontransgenic cloned goat (K03) vs. control goat (S2); **B** *ASMT* transgenic cloned goat (K2020) vs. control goat (S2); **C** nontransgenic cloned goat (K03) vs. *ASMT* transgenic cloned goat (K2020). Note: Pie map reflects the distribution proportion of functional regions on the genome, and different colors represent different functional regions of the genome

Based on the methylation site information, Pearson test was performed on the three samples. The CG type was more correlated than CHH and CHG type (Fig. 8). Functional region annotation was performed for the mCpG loci between samples and the differences among the three samples were concentrated in the intergenic and intronic. The difference between K03 and S2 is mainly located in intronic 40,751,9126 and intergenic 60,894,7062. The difference between K2020 and S2 is mainly located in intronic 40,865,3456 and intergenic 61,052,8405. The difference between K03 and K2020 is mainly located in intronic 40,822,7729 and intergenic 60,986,1613 (Fig. 9).

### Differential DMR detection and annotation

**Fig. 10:**
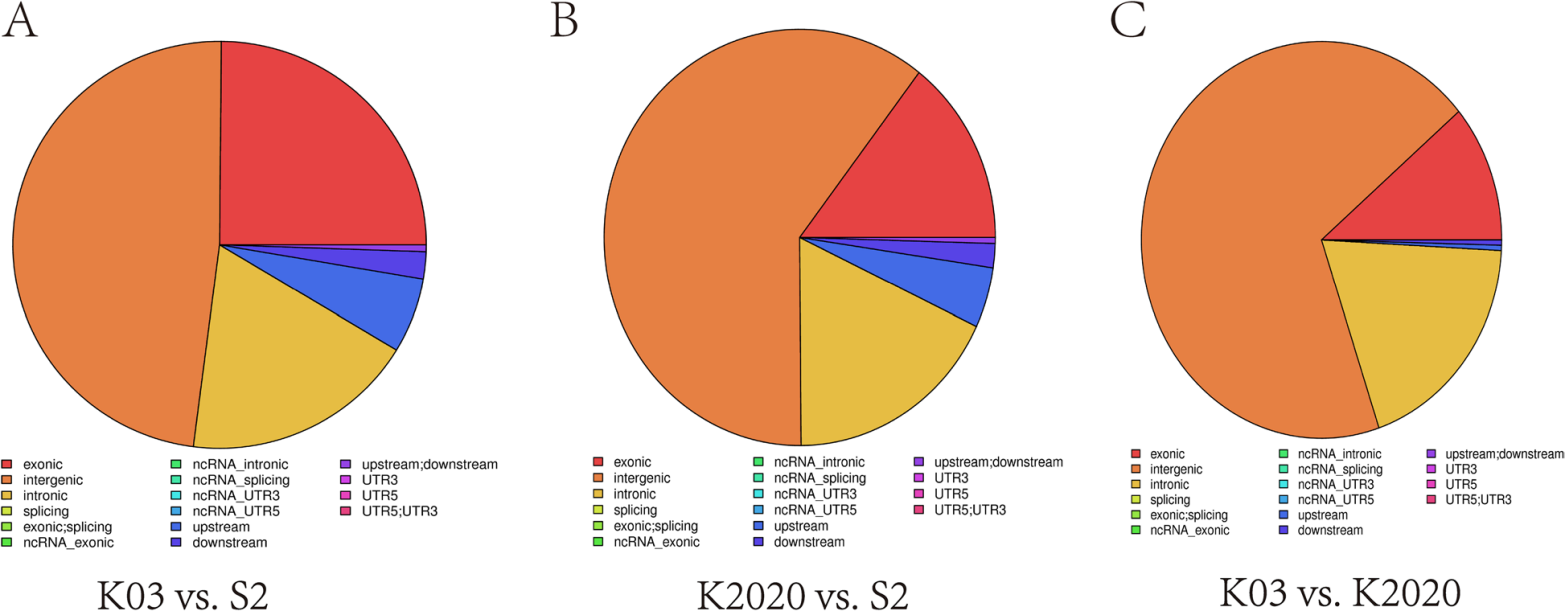
Functional distribution of differential DMR regions. **A** nontransgenic cloned goat (K03) vs. control goat (S2); **B** *ASMT* transgenic cloned goat (K2020) vs. control goat (S2); **C** nontransgenic cloned goat (K03) vs. *ASMT* transgenic cloned goat (K2020). Note: Pie map reflects the distribution proportion of functional regions on the genome, and different colors represent different functional regions of the genome

**Fig. 11:**
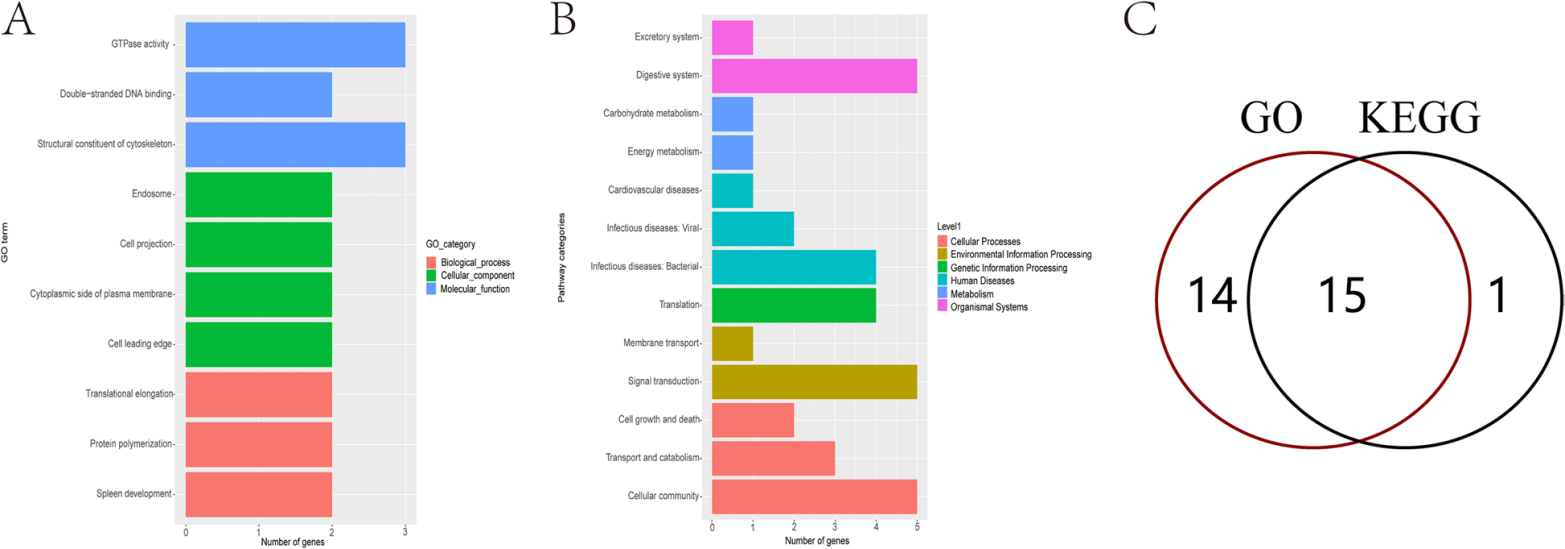
GO/KEGG enrichment analysis of differentially methylated genes in nontransgenic cloned goat (K03) and control goat (S2). **A** GO enrichment analysis; **B** KEGG enrichment analysis; **C** The same genes of GO/KEGG enrichment analysis

**Fig. 12:**
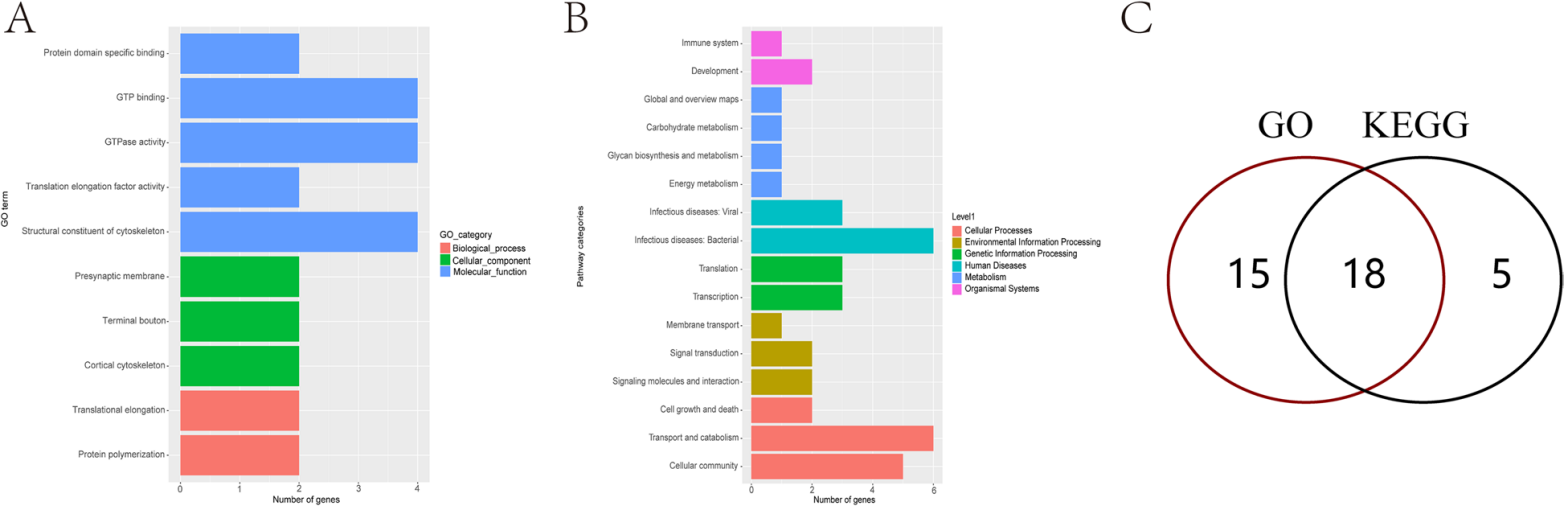
GO/KEGG enrichment analysis of differentially methylated genes in *ASMT* transgenic cloned goat (K2020) and control goat (S2). **A** Go enrichment analysis; **B** KEGG enrichment analysis; **C** The same genes of GO/KEGG enrichment analysis

**Fig. 13:**
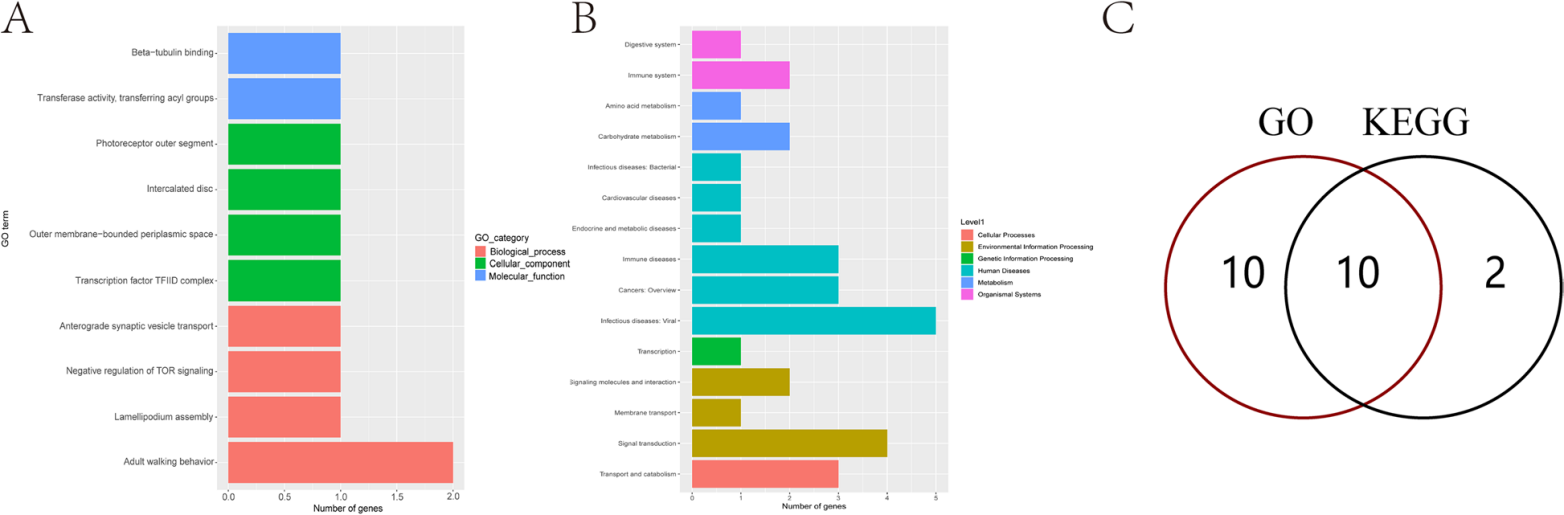
GO/KEGG enrichment analysis of differentially methylated genes in in *ASMT* transgenic cloned goat (K2020) and nontransgenic cloned goat (K03). **A** Go enrichment analysis; **B** KEGG enrichment analysis; **C** The same genes of GO/KEGG enrichment analysis

**Fig. 14:**
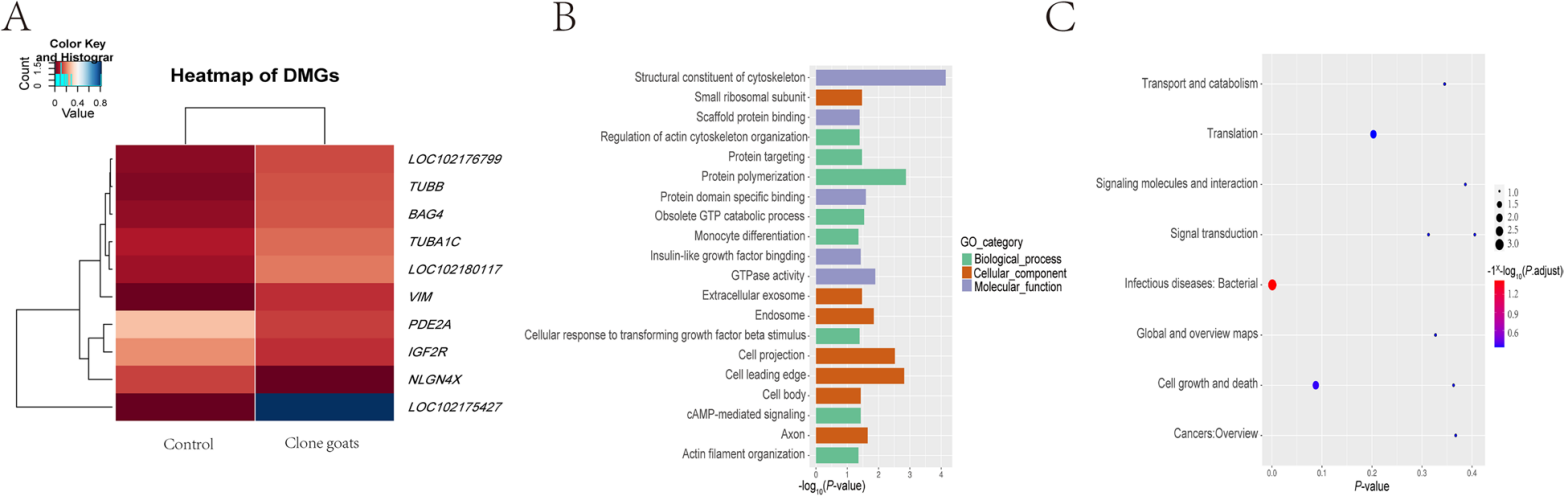
Comparative analysis of differential methylation between cloned goat (*ASMT* transgenic cloned goat (K2020) and nontransgenic cloned goat (K03)) and control goat (S2). **A** Heat map analysis between cloned goat (K2020 and K03) and control goat (S2); **B** Go enrichment analysis between cloned goat (K2020 and K03) and control goat (S2); **C** KEGG enrichment analysis between cloned goat (K2020 and K03) and control goat (S2). Note: There is a scale of 0–0.8 on the heat map. The closer it is to the brown color, the lower the methylation expression level; the closer it is to the blue color, the higher the methylation expression level

**Fig. 15:**
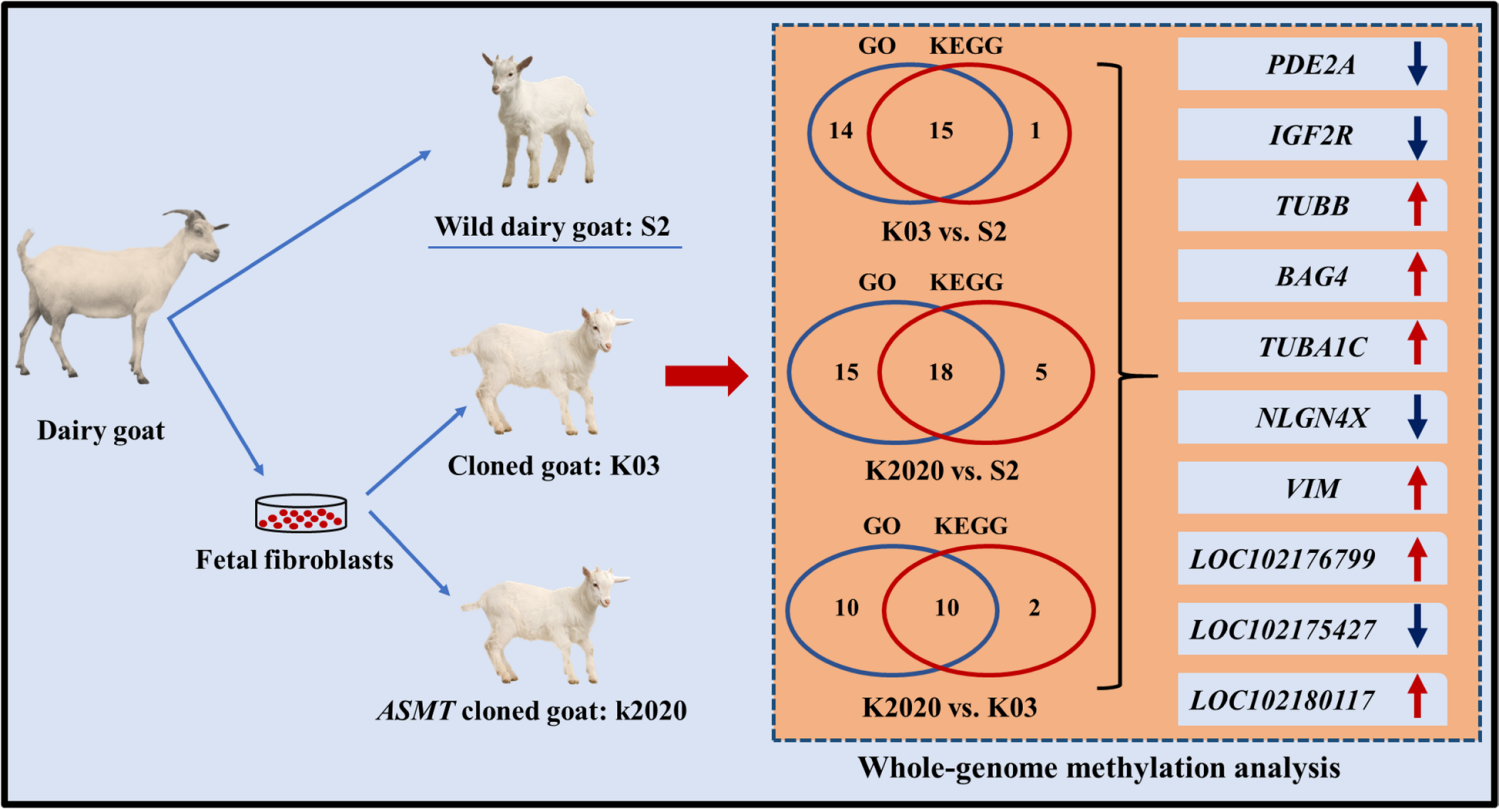
Schematic diagram comparing the results of genome-wide methylation analysis between *ASMT* transgenic cloned goat (K2020), nontransgenic cloned goat (K03) and control goat (S2)

DMR differential methylation, which is an important epigenetic difference, is involved in the regulation of differential gene expression under variable treatments and conditions. Genome-wide assessments were made of differentially methylated regions. The length of DMR region is mainly around 1000 bp (Fig. S6). After functional annotations, we found that the DMR functional regions of the samples were mainly concentrated in the exonic, intergenic and intronic domains. Differences between K2020, K03 and S2 are mainly present in the intergenic functional domain (Fig. 10). GO enrichment analysis was performed in differential DMR regions, mainly focusing on biological-process, cellular-component and molecular-function processes. The DMR regions with differences between K03 and S2 mainly focus on GTPase activity and structural constituent of cytoskeleton pathway, and 29 differential genes were identified (Fig. 11). The DMR regions with differences between K2020 and S2 focus on GTP binding, GTPase activity and structural constituent of cytoskeleton pathway, and 33 differential genes are identified (Fig. 12). Differential DMR between K2020 and K03 mainly focused on adult walking behavior pathway, and 20 differential genes were identified (Fig. 13). At the same time, KEGG analysis of the three samples was enriched in cellular processes, environmental information processing, genetic information processing and human disease, metabolism, organismal systems biological processes. K03 and S2 mainly focused on the digestive system, signal transduction, and cellular community pathways, and 16 different genes were identified (Fig. 11). K2020 and S2 mainly focused on infectious diseases: bacterial, transport and catabolism, and cellular community pathway, and identified 23 differential genes (Fig. 12). K03 and K2020 mainly focus on infectious diseases: viral, signal transduction pathway, and 12 genes were identified (Fig. 13). The 10 common genes related to growth, development and metabolism were found in GO and KEGG enrichment analysis of clone goats and controls. The methylation expression levels of these 10 genes were significantly different between cloned and WT goats, and the expression levels of *NLGN4X* and *LOC1021175427* in cloned goats were significantly higher than that in WT goats. (Fig. 14A). The pathways/terms related to 10 genes were extracted and most notably structural constituent of cytoskeleton (Fig. 14B). The most significant in KEGG enrichment analysis was the infectious diseases bacterial pathway (Fig. 14C). A specific description of the 10 differentially methylated genes is shown in Fig. 15. *BAG4*, *NLGN4X* and *PDE2A* were enriched in environmental information processing. *IGF2R*, *LOC102180117* and *TUBA1C* were enriched in Cellular Processes. *TUBB* and *VIM* were enriched in Human Diseases. *LOC1021175427* was enriched into Genetic Information processing. *LOC102176799* was enriched in Metabolism. *IGF2R* is an imprinted gene and plays an important role in DNA epigenetic modification.

## Discussion

In the current study, to identify the potentially epigenetic variations between the transgenic goats and their wild type made by the SCNT and *ASMT* overexpressed goats with the help of this technology were generated. ASMT is the rate limiting enzyme of melatonin synthesis. Melatonin is an important reproductive regulator which promotes the development of mammalian oocytes and embryos [32], thus we select this gene to avoid any obvious shortcoming to influence the embryo development which would mask the side effects of SCNT on embryo. Melatonin can be synthesized in the mitochondria of oocytes and this locally produced melatonin has the capacity to protect DNA from oxidative damage and improves oocyte quality [33]. Previous study has reported that the ovulation efficiency and lambing rate were improved in the progeny of *AANAT* (another rate limiting enzyme of melatonin synthesis) overexpressed sheep [34]. Thus, we believe that *ASMT* overexpressed goats would have high melatonin level in their ovaries to improve their fecundity. As we know that *GDF9* is expressed in oocytes, and its SNP polymorphism is significantly correlated with fertility [35]. Therefore, in this study, GDF9-ASMT vector was constructed to promote *ASMT* overexpression in oocytes. In addition, to increase the successful rate, the CRISPR/Cas9 method is also used in the study, due to its simple operation and high target efficiency [36]. It has been reported that use of CRISPR/Cas9 to edit *MSTN* gene in *Bamaxiang* pigs promotes their growth rate and muscle fiber proliferation [37]. Application of CRISPR/cas9 system to knock out *FGF5* in Duper sheep increases their hair follicle density [38], in cashmere goats, improves the cashmere yield and fiber length [39]. Thus, in the current study, *FGF5* was also used as the target to produce *ASMT* gene knock-in dairy goats.

It is well documented that DNA methylation is a common epigenetic modification in mammals that affects oocyte meiotic maturation and embryonic development [40]. During embryonic development, DNA methylation will undergo dynamic changes of demethylation and re-methylation [41]. Majority of the cloned embryos stop to development at different stages due to activation failure associated with abnormal methylation [42]. DNA methylation, imprinting and X chromosome inactivation during SCNT embryo reprogramming have drawn a great attention of researchers [43]. The abnormal DNA methylation including elevation of 5-MC, H3K4Me3 and H3K9Me3 may result in reprogramming failure of goat cloned embryos during ZGA [44]. In the current study, we have not found any significant difference at the level of methylation on the chromosome level between cloned and WT animals, which indicates that there were no significant epigenetic modifications between the normally born cloned and WT goats. However, the sequence characteristics of CHG and CHH among the tested goats were significantly different. For instances, in K2020, two side sequence of the CHH extended position site 1 and 9, respectively and the base at sites 1 and 9 was T and the middle site of CHG was A, T, C in sequence. While in K03 and S2, the middle site of CHG was A, C, T in sequence. The results are in consistent with Wang et al. who reported the similar result in the blood and ear tissues of donor and cloned pigs [45]. In addition, the methylation rate of mCpG among different chromosomes also showed some variations. The results of global DNA methylation patterns of fetal and adult muscle development in Hu sheep showed that the methylation levels in the CG context were higher than those in the CHG and CHH contexts [46]. This result is in agreement with our observation. The introns occupy a large proportion of the genes, which affect gene expression by regulating transcription rate and stability [47]. Results of genome-wide methylation in adolescent goats showed that DNA methylation of the promoter was negatively correlated with lncRNA during puberty onset, and methylation regulated the initiation of puberty via lncRNA [48]. In this study, we have observed that the different mCpG loci are mainly present in the intergenic and intronic locations between the cloned and WT animals. Differential methylation regions (DMRs) are involved in the regulation of differential gene expression [49]. Study on genome-wide methylation of Tan sheep show that their *DMGs, KRT71* and *CD44* were highly methylated in mon1, and *ROR2* and *ZDHHC13* were highly methylated in mon48 [50].

In this study, we also analyzed the regional differential genes of DMR. GO enrichment analysis showed that the differential genes were mainly associated to in GTPase activity and structural constituent of cytoskeleton. Furthermore, genes in GO enrichment and KEGG enrichment pathways have identified 10 differentially methylated genes between cloned and WT dairy goats. They belong to different pathways. Among them *IGF2R* is an imprinted gene widely present in mammals [51]. Meng et al. evaluated DNA methylation of living and dead cloned goats and found that DMRs methylation states of *H19* and *IGF2R* were different between each other [52]. In this study, the variations of *IGF2R* gene methylation were found between cloned and WT goats. CNV variation of *BAG4* gene is significantly correlated with sheep growth traits and is an important marker for molecular breeding [53]. *VIM* is involved in the regulation of hair follicle growth cycle by affecting the outer root sheath [54]. *PDE2A* [55], *TUBB* [56] and *TUBA1C* [57] are household genes of mammalian cell structure and metabolism, which affect body development and disease conditions. We observed all these genes have the significantly increased DNA methylation patterns in cloned goats compared to the WT. The expression levels of *NLGN4X* and *LOC1021175427* in cloned goat were significantly higher than that in WT goats. But the expression levels of *VIM* and *TUBB* in WT goats were significantly higher than that in cloned goats. These differences may be related to the somatic cell nuclear transfer technology which impacts the growth and development of cloned animals. Revealing the differential methylation of DNA is of great significance for studying the epigenetic modification of cloned animals which created by different methodologies.

## Conclusion

In this study, we successfully produced *ASMT* ovarian overexpression transgenic goats with the help of SCNT. Whole-genome methylation shows that the differential mCpG sites are mainly present in the intergenic and intronic regions, and the differential DMR length is located around 1000 bp. 56 and 36 DMGs were identified by GO and KEGG analysis in differential DMR, respectively. 10 genes related to growth and development were identified by GO and KEGG enrichment analysis between cloned and WT goats. The significant variations of the differential methylated genes between the cloned and WT animals may be made by the SCNT and these variations may also be response to the relatively low survival rate of the cloned animals compared to their WT controls. The other biological significances of this differentiation warrant further research.

## Supplementary Information

**Additional file 1: Fig. S1.** GDF9-ASMT eukaryotic expression vector.

**Additional file 2: Fig. S2.** Methylation sequencing quality and statistical analyses (A, *ASMT* transgenic cloned goat (K2020); B, control goat (S2); C, Nontransgenic cloned goat (K03)).

**Additional file 3: Fig. S3.** Genome-wide distribution of mean methylation rates (A, *ASMT* transgenic cloned goat (K2020); B, control goat (S2); C, Nontransgenic cloned goat (K03)).

**Additional file 4: Fig. S4.** Density distribution of methylation in functional zones (A, *ASMT* transgenic cloned goat (K2020); B, control goat (S2); C, Nontransgenic cloned goat (K03)).

**Additional file 5: Fig. S5.** mCpG bits cover the depth distribution frequency map (A, *ASMT* transgenic cloned goat (K2020); B, control goat (S2); C, Nontransgenic cloned goat (K03)).

**Additional file 6: Fig. S6.** mCpG bits methylation rate distribution frequency graph (A, *ASMT* transgenic cloned goat (K2020); B, control goat (S2); C, Nontransgenic cloned goat (K03)).

**Additional file 7: Fig. S7.** distribution of different DMR length (A, *ASMT* transgenic cloned goat (K2020); B, control goat (S2); C, Nontransgenic cloned goat (K03)).

## Data Availability

Additional data may be obtained by contacting the corresponding author.

## Abbreviations

AANAT: Aralkylamine N-acetyltransferase
ASMT: Acetylserotonin-O-methyltransferase
DMGs: Differentially methylated genes
DMR: Differentially methylated region
PF: Pass Filter Data
SCNT: Somatic cell nuclear transfer
WGBS: Whole-genome bisulfite sequencing
WT: Wild type
ZGA: Zygotic genome activation

## References

[1] Scheerlinck JPY, Snibson KJ, Bowles VM, Sutton P (2008). Biomedical applications of sheep models: from asthma to vaccines. Trends Biotechnol.

[2] Pinnapureddy AR, Stayner C, McEwan J, Baddeley O, Forman J, Eccles MR (2015). Large animal models of rare genetic disorders: sheep as phenotypically relevant models of human genetic disease. Orphanet J Rare Dis.

[3] Kalds P, Zhou S, Cai B, Liu J, Wang Y, Petersen B (2019). Sheep and goat genome engineering: from random transgenesis to the crispr era. Front Genet.

[4] Wilmut I, Taylor J (2018). Cloning after Dolly. Cell Reprogram.

[5] Bhat SA, Malik AA, Ahmad SM, Shah RA, Ganai NA, Shafi SS (2017). Advances in genome editing for improved animal breeding: a review. Vet World.

[6] Deng S, Li G, Zhang J, Zhang X, Cui M, Guo Y (2013). Transgenic cloned sheep overexpressing ovine toll-like receptor 4. Theriogenology..

[7] Tao J, Yang M, Wu H, Ma T, He C, Chai M (2018). Effects of AANAT overexpression on the inflammatory responses and autophagy activity in the cellular and transgenic animal levels. Autophagy.

[8] Liu Z, Cai Y, Wang Y, Nie Y, Zhang C, Xu Y (2018). Cloning of macaque monkeys by somatic cell nuclear transfer. Cell.

[9] Simmet K, Wolf E, Zakhartchenko V (2020). Manipulating the epigenome in nuclear transfer cloning: where, when and how. Int J Mol Sci.

[10] Samiec M, Skrzyszowska M (2018). Can reprogramming of overall epigenetic memory and specific parental genomic imprinting memory within donor cell-inherited nuclear genome be a major hindrance for the somatic cell cloning of mammals? – a review. Ann N Y Acad Sci.

[11] Skrzyszowska M, Samiec M (2021). Generating cloned goats by somatic cell nuclear transfer—molecular determinants and application to transgenics and biomedicine. Int J Mol Sci.

[12] Matoba S, Zhang Y (2018). Somatic cell nuclear transfer reprogramming: mechanisms and applications. Cell Stem Cell.

[13] Zhang X, Gao S, Liu X (2021). Advance in the role of epigenetic reprogramming in somatic cell nuclear transfer-mediated embryonic development. Stem Cells Int.

[14] Jones PA, Takai D (2001). The role of dna methylation in mammalian epigenetics. Science.

[15] Moore LD, Le T, Fan G (2013). DNA methylation and its basic function. Neuropsychopharmacology.

[16] Jones PA (2012). Functions of DNA methylation: islands, start sites, gene bodies and beyond. Nat Rev Genet.

[17] Zhou C, Wang Y, Zhang J, Su J, An Q, Liu X (2019). H3k27me3 is an epigenetic barrier while kdm6a overexpression improves nuclear reprogramming efficiency. FASEB J.

[18] Gao R, Wang C, Gao Y, Xiu W, Chen J, Kou X (2018). Inhibition of aberrant DNA re-methylation improves post-implantation development of somatic cell nuclear transfer embryos. Cell Stem Cell.

[19] Xu W, Li H, Zhang M, Shi J, Wang Z (2020). Locus-specific analysis of dna methylation patterns in cloned and in vitro fertilized porcine embryos. J Reprod Dev.

[20] Deng M, Zhang G, Cai Y, Liu Z, Zhang Y, Meng F (2020). DNA methylation dynamics during zygotic genome activation in goat. Theriogenology.

[21] Rath MF, Coon SL, Amaral FG, Weller JL, Møller M, Klein DC (2016). Melatonin synthesis: acetylserotonin o-methyltransferase (ASMT) is strongly expressed in a subpopulation of pinealocytes in the male rat pineal gland. Endocrinology.

[22] Martin M (2011). Cutadapt removes adapter sequences from high-throughput sequencing reads. EMBnet J.

[23] Krueger F, Andrews SR (2011). Bismark: a flexible aligner and methylation caller for bisulfite-seq applications. Bioinformatics.

[24] Akalin A, Kormaksson M, Li S, Garrett-Bakelman FE, Figueroa ME, Melnick A (2012). Methylkit: a comprehensive r package for the analysis of genome-wide dna methylation profiles. Genome Biol.

[25] Li H, Handsaker B, Wysoker A, Fennell T, Ruan J, Homer N (2009). 1000 genome project data processing subgroup. The sequence alignment/map format and samtools. Bioinformatics.

[26] Wang K, Li M, Hakonarson H (2010). Annovar: functional annotation of genetic variants from high-throughput sequencing data. Nucleic Acids Res.

[27] Wang Z, Li X, Jiang Y, Shao Q, Liu Q, Chen B (2015). Swdmr: a sliding window approach to identify differentially methylated regions based on whole genome bisulfite sequencing. PLoS One.

[28] Ashburner M, Ball CA, Blake JA, Botstein D, Butler H, Cherry JM (2000). Gene ontology: tool for the unification of biology. Nat Genet.

[29] Consortium GO (2015). Gene ontology consortium: going forward. Nucleic Acids Res.

[30] Kanehisa M, Goto S, Sato Y, Kawashima M, Furumichi M, Tanabe M (2014). Data, information, knowledge and principle: back to metabolism in kegg. Nucleic Acids Res.

[31] Kanehisa M, Goto S (2000). Kegg: Kyoto encyclopedia of genes and genomes. Nucleic Acids Res.

[32] Li Y, Liu H, Wu K, Liu H, Huang T, Chen ZJ (2019). Melatonin promotes human oocyte maturation and early embryo development by enhancing clathrin-mediated endocytosis. J Pineal Res.

[33] He C, Wang J, Zhang Z, Yang M, Li Y, Tian X (2016). Mitochondria synthesize melatonin to ameliorate its function and improve mice oocyte’s quality under in vitro conditions. Int J Mol Sci.

[34] Tian X, Lv D, Ma T, Deng S, Yang M, Song Y (2018). AANAT transgenic sheep generated via ops vitrified-microinjected pronuclear embryos and reproduction efficiency of the transgenic offspring. PeerJ.

[35] Kona S, Chakravarthi VP, Kumar AS, Srividya D, Padmaja K, Rao V (2016). Quantitative expression patterns of *Gdf9* and *Bmp15* genes in sheep ovarian follicles grown in vivo or cultured in vitro. Theriogenology.

[36] Gupta D, Bhattacharjee O, Mandal D, Sen MK, Dey D, Dasgupta A (2019). Crispr-Cas9 system: a new-fangled dawn in gene editing. Life Sci.

[37] Zhu XX, Zhan QM, Wei YY, Yan AF, Feng J, Liu L (2020). Crispr/Cas9-mediated mstn disruption accelerates the growth of chinese bama pigs. Reprod Domest Anim.

[38] Zhang R, Li Y, Jia K, Xu X, Li Y, Zhao Y, et al. Crosstalk between androgen and Wnt/β-catenin leads to changes of wool density in FGF5-knockout sheep. Cell Death Dis. 2020;11(5):407. 10.1038/s41419-020-2622-x PMC7260202 32472005

[39] Hu X, Hao F, Li X, Xun Z, Gao Y, Ren B (2021). Generation of vegf knock-in cashmere goat via the CRISPR/Cas9 system. Int J Biol Sci.

[40] Zeng Y, Chen T (2019). DNA methylation reprogramming during mammalian development. Genes.

[41] Guo H, Zhu P, Yan L, Li R, Hu B, Lian Y (2014). The DNA methylation landscape of human early embryos. Nature.

[42] Yamazaki T, Hatano Y, Taniguchi R, Kobayashi N, Yamagata K (2020). Editing DNA methylation in mammalian embryos. Int J Mol Sci.

[43] Niemann H (2016). Epigenetic reprogramming in mammalian species after scnt-based cloning. Theriogenology.

[44] Deng M, Liu Z, Chen B, Wan Y, Yang H, Zhang Y (2020). Aberrant DNA and histone methylation during zygotic genome activation in goat cloned embryos. Theriogenology.

[45] Wang M, Feng S, Ma G, Miao Y, Zuo B, Ruan J (2020). Whole-genome methylation analysis reveals epigenetic variation in cloned and donor pigs. Front Genet.

[46] Fan Y, Liang Y, Deng K, Zhang Z, Zhang G, Zhang Y (2020). Analysis of dna methylation profiles during sheep skeletal muscle development using whole-genome bisulfite sequencing. BMC Genomics.

[47] Shaul O (2017). How introns enhance gene expression. Int J Biochem Cell Biol.

[48] Yang C, Gao X, Ye J, Ding J, Liu Y, Liu H (2018). The interaction between DNA methylation and long non-coding RNA during the onset of puberty in goats. Reprod Domest Anim.

[49] Srivastava A, Karpievitch YV, Eichten SR, Borevitz JO, Lister R (2019). Home: a histogram based machine learning approach for effective identification of differentially methylated regions. BMC Bioinformatics.

[50] Liu Y, Xu Q, Kang X, Wang K, Wang J, Feng D (2021). Dynamic changes of genomic methylation profiles at different growth stages in chinese tan sheep. J Anim Sci Biotechnol.

[51] Vu T, Jirtle R, Hoffman A (2006). Cross-species clues of an epigenetic imprinting regulatory code for the *IGF2R* gene. Cytogenet Genome Res.

[52] Meng L, Wan Y, Sun Y, Zhang Y, Wang Z, Song Y (2013). Generation of five human lactoferrin transgenic cloned goats using fibroblast cells and their methylation status of putative differential methylation regions of *IGF2R* and *H19* imprinted genes. PLoS One.

[53] Yang Z, Cao X, Ma Y, Cheng J, Song C, Jiang R (2021). Novel copy number variation of the *BAG4* gene is associated with growth traits in three chinese sheep populations. Anim Biotechnol.

[54] Rile N, Liu Z, Gao L, Qi J, Zhao M, Xie Y (2018). Expression of vimentin in hair follicle growth cycle of inner mongolian cashmere goats. BMC Genomics.

[55] Doummar D, Dentel C, Lyautey R, Metreau J, Keren B, Drouot N (2020). Biallelic pde2a variants: a new cause of syndromic paroxysmal dyskinesia. Eur J Hum Genet.

[56] Li D, Shen KM, Zackai EH, Bhoj EJ (2020). Clinical variability of tubb-associated disorders: diagnosis through reanalysis. Am J Med Genet A.

[57] Albahde MAH, Zhang P, Zhang Q, Li G, Wang W (2020). Upregulated expression of tuba1c predicts poor prognosis and promotes oncogenesis in pancreatic ductal adenocarcinoma via regulating the cell cycle. Front Oncol.

