# Supplementary figures and images for: Whole-genome methylation analysis reveals epigenetic variation between wild-type and nontransgenic cloned, ASMT transgenic cloned dairy goats generated by the somatic cell nuclear transfer
Source: J Anim Sci Biotechnol. 2022 Nov 25;13:145. doi: 10.1186/s40104-022-00764-6 (PMC9701027; doi:10.1186/s40104-022-00764-6)

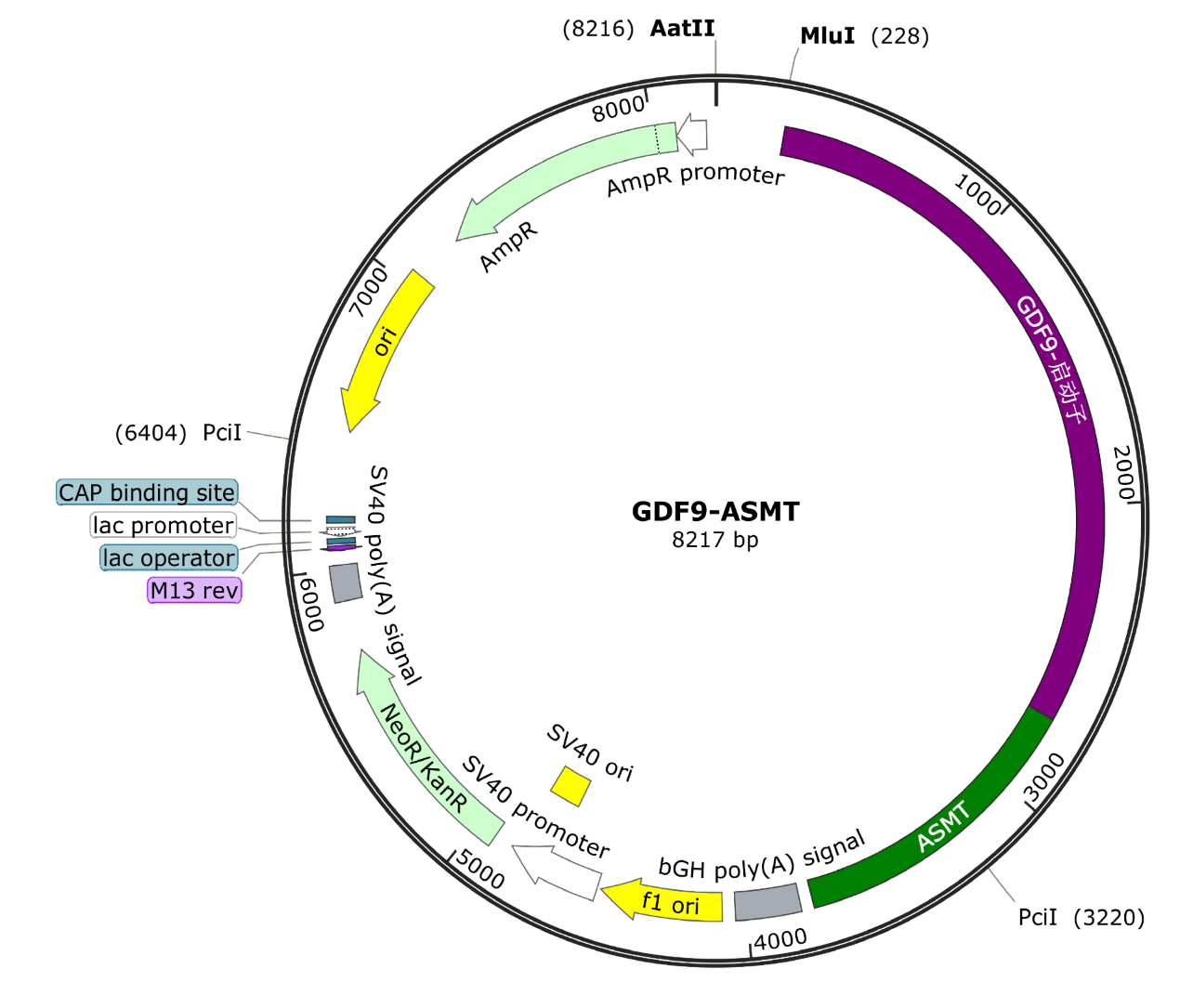


**Fig. S1** GDF9-ASMT eukaryotic expression vector

Supplement: Supplementary file 1 — Additional file 1: Fig. S1. GDF9-ASMT eukaryotic expression vector. [file 40104_2022_764_MOESM1_ESM.docx]
