# Supplementary material for: Whole-genome methylation analysis reveals epigenetic variation between wild-type and nontransgenic cloned, ASMT transgenic cloned dairy goats generated by the somatic cell nuclear transfer
Source: J Anim Sci Biotechnol. 2022 Nov 25;13:145. doi: 10.1186/s40104-022-00764-6 (PMC9701027; doi:10.1186/s40104-022-00764-6)
Supplement: Supplementary file 6 — Additional file 6: Fig. S6. mCpG bits methylation rate distribution frequency graph (A, ASMT transgenic cloned goat (K2020); B, control goat (S2); C, Nontransgenic cloned goat (K03)). [file 40104_2022_764_MOESM6_ESM.docx]

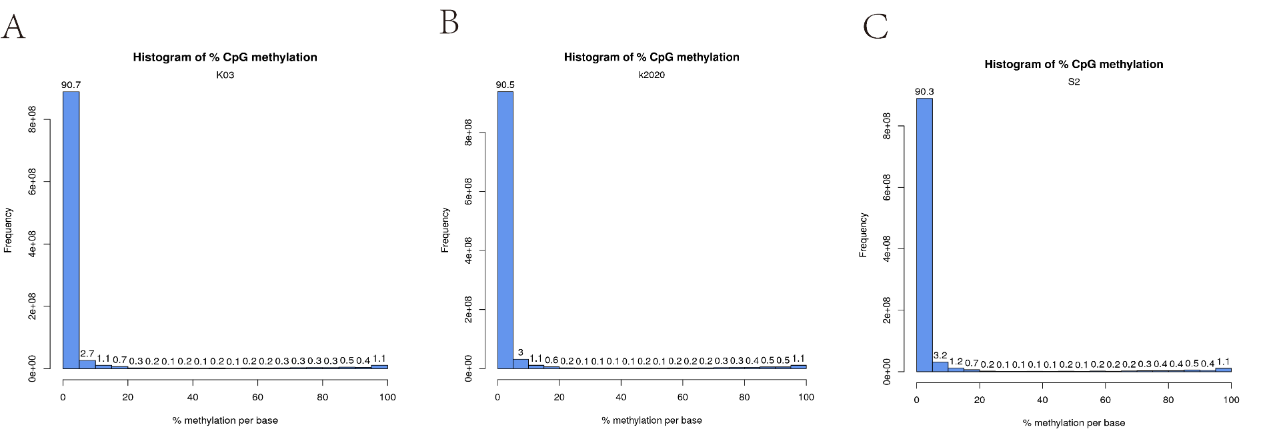


**Fig. S6** mCpG bits methylation rate distribution frequency graph. **A**, *ASMT* transgenic cloned goat (K2020); **B**, control goat (S2); **C**, Nontransgenic cloned goat (K03). The horizontal coordinates are the methylation rate of the mCpG bits, and the ordinates are the total number of mCpG bits at the specified methylation rate, and the scale is marked
