# Supplementary material for: Whole-genome methylation analysis reveals epigenetic variation between wild-type and nontransgenic cloned, ASMT transgenic cloned dairy goats generated by the somatic cell nuclear transfer
Source: J Anim Sci Biotechnol. 2022 Nov 25;13:145. doi: 10.1186/s40104-022-00764-6 (PMC9701027; doi:10.1186/s40104-022-00764-6)
Supplement: Supplementary file 7 — Additional file 7: Fig. S7. distribution of different DMR length (A, ASMT transgenic cloned goat (K2020); B, control goat (S2); C, Nontransgenic cloned goat (K03)). [file 40104_2022_764_MOESM7_ESM.docx]

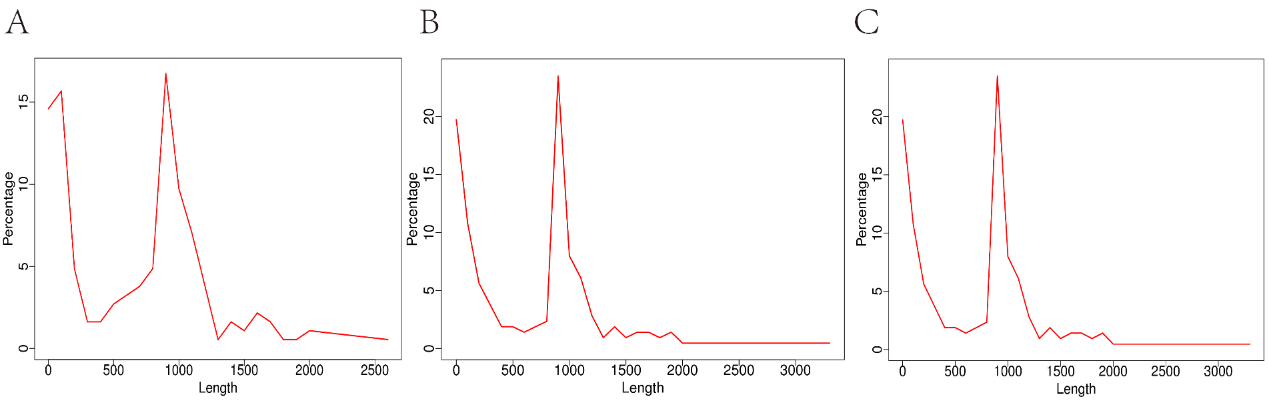


**Fig. S7** distribution of different DMR length. **A**, *ASMT* transgenic cloned goat (K2020); **B**, control goat (S2); **C**, Nontransgenic cloned goat (K03). The horizontal coordinate represents the area length; the ordinate represents the number of DMRs within the specified area length range
